# Draft Genome Sequence of *Fonsecaea nubica* Strain CBS 269.64, Causative Agent of Human Chromoblastomycosis

**DOI:** 10.1128/genomeA.00735-16

**Published:** 2016-08-04

**Authors:** Flávia F. Costa, Sybren de Hoog, Roberto T. Raittz, Vinicius A. Weiss, Aniele C. R. Leão, Amanda Bombassaro, Jiufeng Sun, Leandro F. Moreno, Emanuel M. Souza, Fabio O. Pedrosa, Maria Berenice R. Steffens, Valter Baura, Michele Z. Tadra-Sfeir, Eduardo Balsanelli, M. Javad Najafzadeh, Renata R. Gomes, Maria S. Felipe, Marcus Teixeira, Germana D. Santos, Liyan Xi, Mauro Antônio Alves de Castro, Vânia A. Vicente

**Affiliations:** a Engineering Bioprocess and Biotechnology Post-Graduation Program, Department of Bioprocess Engineering and Biotechnology, Federal University of Paraná, Curitiba, Paraná, Brazil; b Microbiology, Parasitology and Pathology Post-Graduation Program, Department of Pathology, Federal University of Paraná, Curitiba, Paraná, Brazil; c Laboratory of Bioinformatics, Professional and Technological Education Sector, Federal University of Paraná, Curitiba, Paraná, Brazil; d Institute of Public Health, Guangdong Provincial Center for Disease Control and Prevention, Guangdong, China; e CBS-KNAW Fungal Biodiversity Center, Utrecht, The Netherlands; f Department of Biochemistry, Federal University of Paraná, Curitiba, Paraná, Brazil; g Department of Parasitology and Mycology, School of Medicine, Mashhad University of Medical Sciences, Mashhad, Iran; h Catholic University of Brasilia UCB, Brasilia, Brazil; i Division of Pathogen Genomics, Translational Genomics Research Institute, Flagstaff, Arizona, USA; j Department of Dermatology, Sun Yat-Sen Memorial Hospital, Sun Yat-sen University, Guangzhou, China

## Abstract

On the basis of multilocus phylogenetic data, *Fonsecaea nubica* was described in 2010 as a molecular sibling of *F. monophora*, an established agent of the human skin disease chomoblastomycosis in tropical zones. Genome analysis of these pathogens is mandatory to identify genes involved in the interaction with host and virulence.

## GENOME ANNOUNCEMENT

The genus *Fonsecaea* comprises anamorph members in the *Chaetothyriales*, an ascomycete order of black yeasts and filamentous relatives covering numerous opportunistic pathogens on humans (1–3). *Fonsecaea* is one of the prevalent genera of etiologic agents of chromoblastomycosis (4, 5), a chronic, cutaneous, and subcutaneous infection characterized by slowly expanding, polymorphic skin lesions with muriform cells in tissue, provoking a granulomatous immune response (6, 7). The disease occurs preferentially in humans, although some cases have been reported in other mammals (8–10). Several *Fonsecaea* species are involved as etiologic agents of the disease, i.e., *F. pedrosoi*, *F. monophora*, *F. nubica*, and *F. pugnacius*, each with different virulence potentials (6, 11). *F. nubica* was first described in 2010 with type strain CBS 269.64, isolated from a human patient with chromoblastomycosis in west Cameroon (6, 12). Genome analysis of the pathogenic fungus *F. nubica* is needed to identify genes involved in the interaction with host cells and molecular mechanisms in response to cytotoxic agents (13).

Strain *F. nubica* CBS 269.64 was grown in Sabouraud’s broth, with shaking at 150 rpm at 28°C for 7 days and DNA was extracted by the cetyltrimethylammonium bromide (CTAB) method with phenol-chloroform/isoamyl alcohol. Total DNA was purified with the microbial DNA UltraClean kit. DNA of *F. nubica* was used for library construction using the Ion Plus Fragment library kit (Thermo, FisherScientific) and Nextera XT (Illumina) following the manufacturer’s instructions. The libraries were sequenced on an Ion Proton (Thermo, FisherScientific) for single-end reads and in Miseq (Illumina) for paired-end reads. The quality of the reads was assessed by means of FastQC (http://www.bioinformatics.babraham.ac.uk/projects/fastqc). The reads were assembled *de novo* using SPADES v3.6.2 (14). The draft genome comprised 258 contigs and the genome size was 33.7 Mb, with a G+C content of 52.46%. Protein-coding genes were predicted with GeneMark-ES (15). Gap closure was performed with FGAP software (16). Annotation for 11,681 predicted genes were assigned based on similarity searches against the nr database using RAFTS3 (17) and InterProScan (18) comparisons. The genome contained 36 tRNAs identified using ARAGORN (19).

Information about the genome sequence of this black yeast might provide a better understanding of the basic mechanisms of adaptation to requirements of the environmental habitat, and of pathogenicity and virulence.

### Nucleotide sequence accession numbers.

This whole-genome shotgun project has been deposited in DDBJ/ENA/GenBank under accession number LVCJ00000000. The version described in this paper is version LVCJ01000000.

## References

[1] Xi L, Sun J, Lu C, Liu H, Xie Z, Fukushima K, Najafzadeh MJ, De Hoog GS 2009 Molecular diversity of *Fonsecaea* (*Chaetothyriales*) causing chromoblastomycosis in southern China. Med Mycol 47:27–33. doi:10.1080/13693780802468209. 18951291

[2] Hoog GS, Guarro J, Gené J, Figueras MJ 2000 Atlas of clinical fungi. Centraalbureau voor Schimmelcultures/Universitat Rovira i Virgili, Utrecht, the Netherlands.

[3] Badali H, Gueidan C, Najafzadeh MJ, Bonifaz A, Van Den Ende AH, de Hoog GS 2008 Biodiversity of the genus *Cladophialophora*. Stud Mycol 61:175–191. doi:10.3114/sim.2008.61.18. 19287540 PMC2610306

[4] Martinez R, Tovar LJ 2007 Chromoblastomycosis. Clin Dermatolol 25:188–194. doi:10.1016/j.clindermatol.2006.05.007.

[5] Tanabe H, Kawasaki M, Mochizuki T, Ishizaki H 2004 Species identification and strain typing of *Fonsecaea pedrosoi* using ribosomal RNA gene internal transcribed spacer regions. Nippon Ishinkin Gakkai Zasshi 45:105–112. http://doi.org/10.3314/jjmm.45.105. doi:10.3314/jjmm.45.105. 15118668

[6] De Hoog GS, Attili-Angelis D, Vicente VA, Van Den Ende AH, Queiroz-Telles F 2004 Molecular ecology and pathogenic potential of *Fonsecaea* species. Med Mycol 42:405–416. doi:10.1080/13693780410001661464. 15552642

[7] Bonifaz A, Carrasco-Gerard E, Saúl A 2001 Chromoblastomycosis: clinical and mycologic experience of 51 cases. Mycoses 44:1–7. doi:10.1046/j.1439-0507.2001.00613.x. 11398635

[8] Gams W, McGinnis MR 1983 *Phialemonium*, a new anamorph genus intermediate between *phialophora* and *Acremonium*. Mycologia 75:977–987. doi:10.2307/3792653.

[9] Rippon JW 1988 Medical mycology, the pathogenic fungi and pathogenic *Actionmycetes*, 3rd ed. W. B. Saunders, Philadelphia, PA.

[10] De Hoog GS, Queiroz-Telles F, Haase G, Fernandez-Zeppenfeldt G, Attili Angelis D, Gerrits Van Den Ende AH, Matos T, Peltroche-Llacsahuanga H, Pizzirani-Kleiner AA, Rainer J, Richard-Yegres N, Vicente V, Yegres F 2000 Black fungi: clinical and pathogenic approaches. Med Mycol 38:243–250. 11204152

[11] Najafzadeh MJ, Sun J, Vicente VA, Klaassen CH, Bonifaz A, Gerrits Van Den Ende AH, Menken SB, de Hoog GS 2011 Molecular epidemiology of *Fonsecaea* species. Emerg Infect Dis 17:464–469. doi:10.3201/eid1703.100555. 21392438 PMC3165995

[12] Najafzadeh MJ, Sun J, Vicente V, Xi L, van den Ende AH, de Hoog GS 2010 *Fonsecaea nubica* sp. nov., a new agent of human chromoblastomycosis revealed using molecular data. Med Mycol 48:800–806. doi:10.3109/13693780903503081. 20302550

[13] Najafzadeh MJ, Gueidan C, Badali H, Van Den Ende AH, Xi L, de Hoog GS 2009 Genetic diversity and species determination in the opportunistic genus *Fonsecaea*. Med Mycol 47:17–25. doi:10.1080/13693780802527178. 19107635

[14] Bankevich A, Nurk S, Antipov D, Gurevich AA, Dvorkin M, Kulikov AS, Lesin VM, Nikolenko SI, Pham S, Prjibelski AD, Pyshkin AV, Sirotkin AV, Vyahhi N, Tesler G, Alekseyev MA, Pevzner PA 2012 SPAdes: a new genome assembly algorithm and its applications to single-cell sequencing. J Comput Biol 19:455–477. doi:10.1089/cmb.2012.0021. 22506599 PMC3342519

[15] Ter-Hovhannisyan V, Lomsadze A, Chernoff YO, Borodovsky M 2008 Gene prediction in novel fungal genomes using an ab initio algorithm with unsupervised training. Genome Res 18:1979–1990. doi:10.1101/gr.081612.108. 18757608 PMC2593577

[16] Piro VC, Faoro H, Weiss VA, Steffens MB, Pedrosa FO, Souza EM, Raittz RT 2014 FGAP: an automated gap closing tool. BMC Res Notes 7:371. doi:10.1186/1756-0500-7-371. 24938749 PMC4091766

[17] Viale RA, Pedrosa FO, Weiss VA, Guizelini D, Tibaes JH, Marchaukoski JN, Souza EM, Raittz RT 31 5 2016 RAFTS3: rapid alignment free tool for sequences similarity search. bioRxiv. doi:10.1101/055269.

[18] Jones P, Binns D, Chang HY, Fraser M, Li W, McAnulla C, McWilliam H, Maslen J, Mitchell A, Nuka G, Pesseat S, Quinn AF, Sangrador-Vegas A, Scheremetjew M, Yong SY, Lopez R, Hunter S 2014 Inter-ProScan 5: genome-scale protein function classification. Bioinformatics 30:1236–1240. doi:10.1093/bioinformatics/btu031. 24451626 PMC3998142

[19] Laslett D, Canback B 2004 ARAGORN, a program to detect tRNA genes and tmRNA genes in nucleotide sequences. Nucleic Acids Res 32:11–16. doi:10.1093/nar/gkh152. 14704338 PMC373265

